# Predictive Models for the Diagnostic of Human Visceral Leishmaniasis in Brazil

**DOI:** 10.1371/journal.pntd.0001542

**Published:** 2012-02-28

**Authors:** Tália S. Machado de Assis, Ana Rabello, Guilherme L. Werneck

**Affiliations:** 1 Laboratório de Pesquisas Clínicas, Centro de Pesquisas René Rachou, Fundação Oswaldo Cruz (FIOCRUZ), Belo Horizonte, Minas Gerais, Brazil; 2 Departamento de Epidemiologia, Instituto de Medicina Social, Universidade do Estado do Rio de Janeiro, Rio de Janeiro, Brazil; George Washington University, United States of America

## Abstract

**Background and Objectives:**

In Brazil, as in many other affected countries, a large proportion of visceral leishmaniasis (VL) occurs in remote locations and treatment is often performed on basis of clinical suspicion. This study aimed at developing predictive models to help with the clinical management of VL in patients with suggestive clinical of disease.

**Methods:**

Cases of VL (n = 213) had the diagnosis confirmed by parasitological method, non-cases (n = 119) presented suggestive clinical presentation of VL but a negative parasitological diagnosis and a firm diagnosis of another disease. The original data set was divided into two samples for generation and validation of the prediction models. Prediction models based on clinical signs and symptoms, results of laboratory exams and results of five different serological tests, were developed by means of logistic regression and classification and regression trees (CART). From these models, clinical-laboratory and diagnostic prediction scores were generated. The area under the receiver operator characteristic curve, sensitivity, specificity, and positive predictive value were used to evaluate the models' performance.

**Results:**

Based on the variables splenomegaly, presence of cough and leukopenia and on the results of five serological tests it was possible to generate six predictive models using logistic regression, showing sensitivity ranging from 90.1 to 99.0% and specificity ranging from 53.0 to 97.2%. Based on the variables splenomegaly, leukopenia, cough, age and weight loss and on the results of five serological tests six predictive models were generated using CART with sensitivity ranging from 90.1 to 97.2% and specificity ranging from 68.4 to 97.4%. The models composed of clinical-laboratory variables and the rk39 rapid test showed the best performance.

**Conclusion:**

The predictive models showed to be a potential useful tool to assist healthcare systems and control programs in their strategical choices, contributing to more efficient and more rational allocation of healthcare resources.

## Author Summary

Visceral leishmaniasis (VL) is a neglected tropical disease endemic to 65 countries, including Brazil, where the disease frequently occurs in remote locations and treatment is often performed on the basis of clinical suspicion. Predictive models based on scoring systems could be a helpful tool for the clinical management of VL. Based on clinical signs and symptoms, and five different serological tests of 213 patients with parasitologically confirmed (cases) and 119 with clinical suspicion of VL but with another confirmed etiology (non-cases), twelve prediction models using logistic regression and classification and regression trees (CART) for VL diagnosis were developed. The model composed of the clinical-laboratory variables and the rk39 rapid test showed the best performance in both logistic regression and CART (Sensitivity of 90.1% and specificity ranging from 97.2–97.4%). The scoring system is simple and based on the clinical-laboratory findings that are easily available in most clinical settings. The results suggest that those models might be useful in locations where access to available diagnostic methods is difficult, contributing to more efficient and more rational allocation of healthcare resources.

## Introduction

Visceral leishmaniasis (VL) is a neglected tropical disease caused by the intracellular protozoan parasite *Leishmania infantum* (syn. *Leishmania chagasi*). The disease is endemic to 65 countries and 90% of world cases are reported in India, Bangladesh, Nepal, Ethiopia, Brazil, and the Sudan [1]. In Brazil, more then 15.000 VL cases were reported between 2007 and 2010, with 880 deaths [2]. The disease primarily affects the poorest people and is fatal if untreated. The control strategies used in Brazil to reduce the disease morbidity and mortality rates consists on the early diagnosis and treatment of human cases and the control of the populations of domestic reservoirs and vectors [3].

Early diagnosis is a challenge in Brazil, as in other affected countries, where the disease is still frequently treated only on the basis of clinical suspicion. Clinically, the disease is characterized by prolonged fever, substantial weight loss, hepatomegaly, splenomegaly, pancytopenia, hypergammaglobulinemia [3], [4]. The firm diagnosis of VL needs to rely on efficacious laboratorial support. The current reference test for disease diagnostic is the microscopic demonstration of *Leishmania* in spleen, bone marrow, lymph nodes or liver aspirates, but both the aspiration procedure and the reading of slides require a high level of expertise that makes them unsuitable for generalized field use [1], [3].

Several serological diagnostic methods have been widely evaluated for the diagnosis of VL, such as the enzyme linked immunosorbent assay (ELISA) with different antigens and the indirect fluorescence antibody test (IFAT). In Brazil, IFAT is the serologic test made available by the Public Health System. ELISAs and IFAT depend on equipment and laboratorial infrastructure. Two other tests easy to use have been appointed as appropriate for the diagnosis of VL in control programs: the Direct Agglutination Test (DAT) and the rK39 rapid tests [5]–[8].

The development of predictive models could help in the management of patients, especially in towns where the access to diagnostic methods is difficult, being useful as a cost-effective tool in a health care system with limited resources. This study aimed at developing models based on scoring systems using logistic regression and classification and regression trees (CART) to predict the occurrence of VL in patients with suggestive clinical of disease in Brazil.

## Methods

### Settings and patient selection

The models were developed using a database generated from a prospective study conducted in four states of Brasil, published elsewhere [7], [9]. We evaluated a group of 332 patients with symptoms and/or signs suggestive of VL referred for diagnostic and eventual treatment in states of Maranhão (Federal University of Maranhão, 35 patients enrolled), Piauí (Federal University of Piauí, 121 patients), Bahia (Gonçalo Muniz Research Center, 119 patients) and Minas Gerais (René Rachou Research Center, 57 patients), from May 2005 and May 2007.

By the end of clinical investigation, all VL cases had the diagnosis confirmed by parasitological methods. The non-cases had suggestive clinical presentation of VL, a negative parasitological diagnosis and the accomplished diagnosis of another disease. The non-cases were diagnosed with various diseases, such as leukemia, liver disease, schistosomiasis, ascariasis, liver fibrosis, lymphoma, rheumatoid arthritis, malaria, mononucleosis, typhoid fever, marrow aplasia, liver cirrhosis, meningitis, lupus erythematosus, encephalitis, tuberculosis, among others.

### Procedures and diagnostic tests

Patients underwent a standardized interview regarding epidemiological and clinical history and a physical examination. IFAT was performed with an industrial kit (Biomanguinhos, Rio de Janeiro, Brazil) according to the manufacturer's instructions. Samples scored positive when fluorescent microscopy showed clear evidence that they produced a cytoplasmic or membranous fluorescence with promastigotes using a cut-off dilution of 1∶80. *L. chagasi*-ELISA and rK39-ELISA were performed according to Assis et al. (2008) [9]. The cutoff of reactions was determined as the mean plus two standard deviations of the absorbance of control sera (n = 20). DAT was performed according to Pedras et al. (2008) [10]. The cutoff value was determined by analyzing the receiver operator characteristic curve. Rapid test (IT-LEISH® Diamed Latino-America S. A. - Cressier sur Morat, Switzerland) was performed according to the manufacturer's instructions and Assis et al. (2008) [9]. The test was positive when two red lines appeared in the middle of the nitrocellulose membrane, negative when only one redline appeared and invalid when no line was evident. The rapid test and the bone marrow aspirate were performed at the center of origin of the patients evaluated; all other serological tests were performed at the Rene Rachou Research Center.

### Ethical issues

The Research Ethics Committee of René Rachou Research Center and all other institutions involved in this study had previously approved the informed consent forms and procedures. Written informed consent was obtained from all the adults and from minors' parents or legal guardians. The study was conducted in agreement with the principles of the Helsinki Declaration and the Resolution 196/96 of the National Health Council of the Ministry of Health that regulates research involving human subjects in Brazil (CEPSH/CPqRRn°: 13/2003).

### Statistical analysis

The original data set was randomly divided into 2 parts: the “test sample” (patients from Maranhão, Piauí and Minas Gerais, n = 213) was used to construct the models and the “validation sample” (patients from Bahia, n = 119) was used to validate the models. Predictive models were built using logistic regression and CART. Statistical analyses were performed using Stata, version 10.0 (Stata), and Splus, version 4.5 (StatSci).

For developing predictive models with logistic regression, initially the most important factors associated with the occurrence of visceral leishmaniasis were identified. A p-value of ≤0.2 for the univariate association with visceral leishmaniasis was used for selecting variables for the multivariate model. A stepwise elimination procedure was performed, using a p-value de ≤0.05 as the criterion for variables to remain in the model.

A predictive model based on a scoring system, with points allocated to each prognostic factor, was created from the final logistic regression model run in the test sample. The scoring system was generated by dividing the value of the regression coefficient of each variable by the smallest coefficient and rounding the quotients to the closest integer [11]. Posteriorly, the final score was obtained through the sum of points attributed to the presence of each predictive variable that remained in the final model and to the results of five diagnostic methods: IFAT, *L. chagasi*-ELISA, rK39-ELISA, DAT and rapid test.

For constructing predictive model using CART all available variables were initially included in the analysis. The CART method was used to build a binary classification tree through successive partitions, dividing the data into more homogeneous subgroups at each split (“node”). At each node, the algorithm selected the variable with the greatest capacity for discriminating between the 2 outcome groups (VL and non-VL). The first division of the tree corresponds to the variable with the greatest ability to discriminate between VL cases and non-VL patients; the discriminatory power decreases with each subsequent division (“branch”).

The CART algorithm adds nodes until they are homogenous or contains few observations. The problem of creating a useful tree is to find suitable guidelines to achieve a tree with a lower level of misclassification but, at the same time, not too much adjusted to the data. This can be accomplished by downsizing (“pruning”) the tree. The general principle of pruning is that the tree of best size would have the lowest misclassification rate for an individual not included in the original data [12]. Pruning was achieved by decreasing the number of nodes without a significant increase of deviance, with the aid of a graph that shows the relationship between deviance and the number of nodes on the tree [13]. The best tree suggested by our analysis had 7 leaves.

The sensitivity, specificity, positive predictive value (PPV) and area under the receiver operator characteristic (ROC) curve were used to evaluate the performance of the models. The sensitivity is the probability of the test result be positive among patients with the disease, specificity is the probability of the test result be negative among patients without the disease and PPV is the probability that a patient has the disease given a positive test result. The ROC curve consists of a graph of sensitivity versus false positive rate and the area under this curve provides a summary of the ability of a test to discriminate two groups (here, VL and non-VL patients).

## Results

Three hundred thirty-two patients were included in the analysis, 213 parasitologically confirmed VL cases and 119 non-cases with clinical suspicion of VL but with another confirmed etiology. Detailed description of the group and validation of the rK39 rapid test and DAT is reported by Assis et al. (2011) [7]. The average age of the VL cases in test sample was 21 years (1 month to 74 years), and 63% (n = 88) were female and the average age of the non-cases was 16 years (2 months to 66 years), and 60% (n = 44) were female. Table 1 shows the clinical and laboratory characteristics of subjects in the test sample. Table 2 shows the predictive variables that remained in the final logistic regression model: Splenomegaly, leukopenia and cough. The score system generated by using logistic regression attributed −1 point for cough, 1 point for leukopenia, 3 points for splenomegaly and positive IFAT, 4 points for positive *L. chagasi* ELISA, 5 points for positive rK39 rapid test, 6 points for positive rK39-ELISA and 7 points for positive DAT (Table 3).

**Table 1:** Clinical and laboratory characteristics of VL and non-VL cases, among 213 patients with clinical suspicion of VL in test sample.

Clinical and laboratory characteristics	VL cases		Non- LV cases		OR	95% CI	p value
	N	%	N	%			
Age (years)		Continuous			0.9	0.96–0.99	0.001
Sex							
Female	88	62.9	44	60.3	1.0		
Male	52	37.1	29	39.7	0.9	0.50–1.60	0.70
Weight loss							
Yes	115	87.8	51	78.5	2.0	0.90–4.34	0.09
No	16	12.2	14	21.5	1.0		
Cough							
Yes	48	35.6	35	52.2	0.5	0.28–0.91	0.02
No	87	64.4	32	47.8	1.0		
Diarrhea							
Yes	34	25.4	19	28.4	0.9	0.44–1.70	0.6
No	100	74.6	48	71.6	1.0		
Jaundice							
Yes	19	14.3	21	30.4	0.4	0.19–0.77	0.01
No	114	85.7	48	69.6	1.0		
Bleeding							
Yes	13	9.8	15	22.7	0.4	0.16–0.83	0.02
No	120	90.2	51	77.3	1.0	-	-
Splenomegaly							
Yes	127	90.7	36	49.3	10.0	4.83–21.0	<0.001
No	13	9.3	37	50.7	1.0	-	-
Hepatomegaly							
Yes	95	67.9	32	43.8	2.7	1.51–4.84	0.001
No	45	32.1	41	56.2	1.0	-	-
Leukopenia							
Yes	99	74.4	30	42.3	4.0	2.16–7.33	<0.001
No	34	25.6	41	57.7	1.0	-	-
Plaquetopeny							
Yes	81	72.3	34	50.8	2.5	1.35–4.78	0.004
No	31	27.7	33	49.2	1.0	-	-

**Table 2:** Variables significantly associated with visceral leishmaniasis in multiple logistic regression, clinical-laboratory (final model).

Variable	OR	95% CI	p value
Splenomegaly			
Yes	17.0	6.0–47.4	0.00
No	1.00		
Leukopenia			
Yes	4.5	2.0–10.4	0.00
No	1.00		
Cough			
Yes	0.37	0.16–0.84	0.02
No	1.00		

**Table 3:** Predictive performance of different multivariate models in multiple logistic regression.

Models	Variation points	Score cut-off point	Sensitivity (%) (95% CI)	Specificity (%) (95% CI)	Area under ROC Curve (%) (95% CI)	Positive predictive value (%) (95% CI)
1. Clinical-laboratory*	(−1/4)	≥3	81.4 (74.0–88.0)	65.2 (52.4–76.5)	79.4 (72.0–87.0)	82.0 (74.3–88.3)
2. Clinical-laboratory*			91.5 (82.5–97.0)	53.0 (35.5–70.0)	74.0 (61.3–86.3)	79.3 (69.0–87.4)
1. Clinical-laboratory*+*L. chagasi*-ELISA	(−1/8)	≥5	91.5 (85.3–96.0)	80.3 (69.0–89.1)	93.1 (89.5–97.0)	90.1 (84.0–95.0)
2. Clinical-laboratory*+*L. chagasi*-ELISA			90.1 (81.0–96.0)	89.0 (74.0–97.0)	91.0 (85.0–97.3)	94.1 (86.0–98.4)
1. Clinical-laboratory*+IFAT	(−1/7)	≥4	90.0 (83.4–94.5)	77.3 (65.3–87.0)	90.4 (86.0–95.0)	88.5 (82.0–93.4)
2. Clinical-laboratory*+IFAT			99.0 (92.4–100)	78.0 (61.0–90.0)	95.0 (89.0–100)	90.0 (81.0–95.5)
1. Clinical-laboratory*+rK39-ELISA	(−1/10)	≥7	98.0 (93.4–99.5)	88.0 (77.5–95.0)	97.0 (94.1–100)	94.0 (89.0–97.4)
2. Clinical-laboratory*+rK39-ELISA			96.0 (88.1–99.1)	89.0 (74.0–97.0)	93.4 (87.0–100)	94.4 (86.4–98.5)
1. Clinical-laboratory*+DAT	(−1/11)	≥5	90.0 (83.4–94.5)	97.0 (89.5–100)	97.3 (95.4–99.2)	98.3 (94.0–100)
2. Clinical-laboratory*+DAT			91.5 (82.5–97.0)	92.0 (77.5–98.2)	97.0 (88.0–99.1)	94.0 (87.3–100)
1. Clinical-laboratory*+rK39 rapid test	(−1/9)	≥5	94.0 (88.1–97.3)	95.5 (87.3–99.1)	98.5 (97.2–100)	98.0 (93.1–99.5)
2. Clinical-laboratory*+rK39 rapid test			90.1 (81.0–96.0)	97.2 (85.5–100)	95.5 (91.4–99.4)	98.5 (92.0–100)

1 Test sample; 2 Validation sample;

*The model Clinical-laboratory was composed by variables: Splenomegaly and Leukopenia. Points assigned to variables in the models: Cough = −1, leukopenia = 1, splenomegaly and IFAT = 3, *L. chagasi*-ELISA = 4, rK39 rapid test = 5, rK39-ELISA = 6 and DAT = 7.

**Figure 1:**
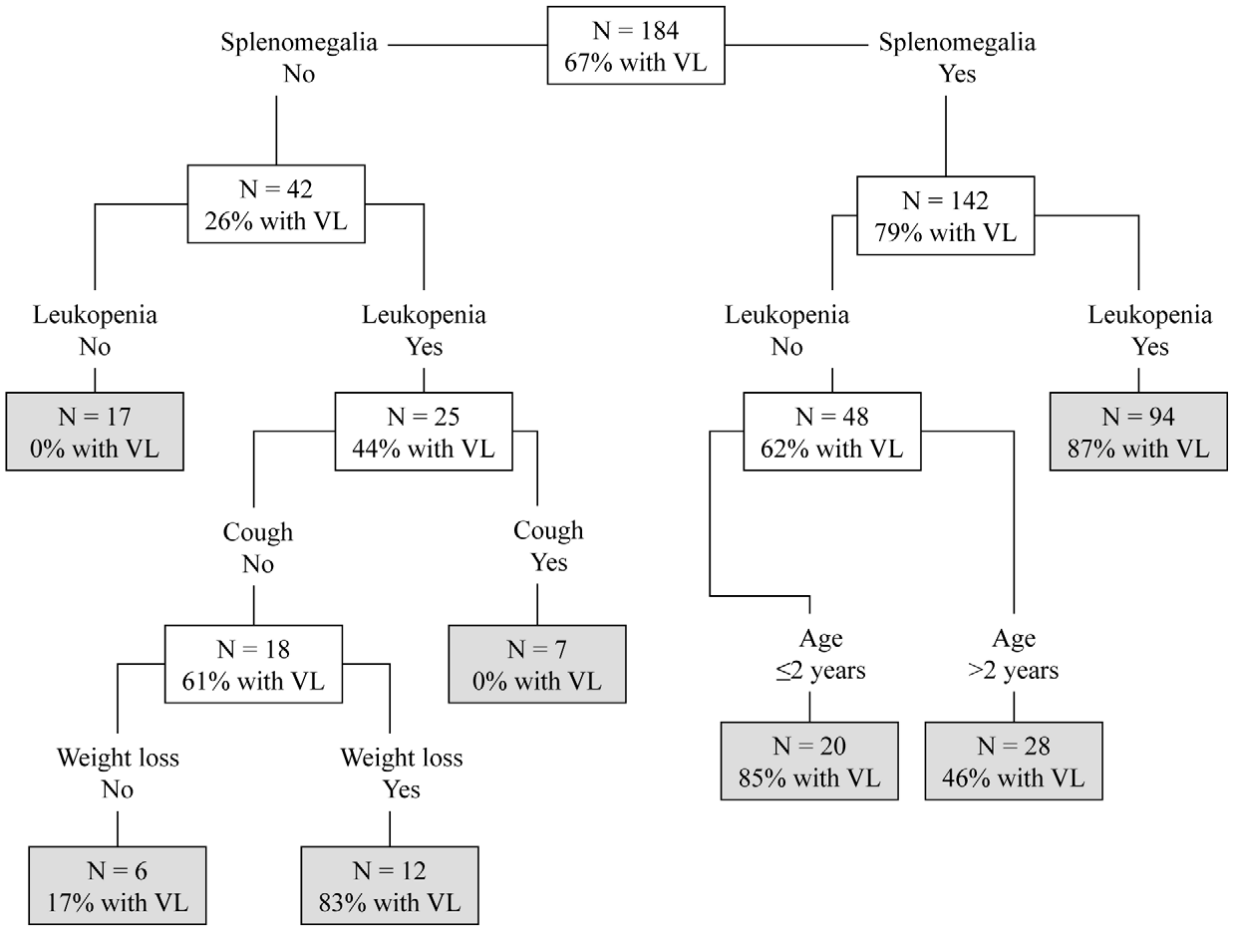
Classification and regression tree for predicting visceral leishmaniasis in patients with suggestive clinical. Classification and regression tree model for predicting VL. The number of patients (N) and the probability of VL (% with VL) are shown at each node. Terminal nodes are shaded.

The CART model was composed by the variables splenomegaly, leukopenia, cough, age and weight loss (Figure 1). The variable with the greatest discriminative power was splenomegaly. The probabilities of VL, as predicted in the leaves of the tree, ranged from 0% to 87%.

Table 3 and 4 show the comparison of the predictive performance of different models generated using logistic regression and CART in terms of the area under the ROC curve, sensitivity, specificity, and PPV evaluated in both test and validation samples. Using logistic regression it was possible to generate predictive models for the diagnostic of VL with sensitivity ranging from 90.1 to 99.0% and specificity ranging from 53.0 to 97.2%. Using CART it was possible to generate predictive models for VL with sensitivity ranging from 90.1 to 97.2% and specificity ranging from 68.4 to 97.4%. Logistic regression and CART in the test sample and validation sample had similar performance for most models.

**Table 4:** Predictive performance of different models in classification and regression trees (CART).

Models	Sensitivity (%) (95% CI)	Specificity (%) (95% CI)	Area under ROC Curve (%) (95% CI)	Positive predictive value (%) (95% CI)
1. CART*	80.4 (73.0–87.0)	75.4 (63.5–85.0)	84.0 (76.2–91.3)	86.3 (79.0–92.0)
2. CART*	90.1 (81.0–96.0)	68.4 (51.3–82.5)	86.0 (75.3–96.0)	84.2 (74.0–92.0)
1. CART*+*L. chagasi*-ELISA	92.0 (86.0–96.0)	85.5 (75.0–93.0)	94.0 (90.2–97.4)	92.4 (86.5–96.3)
2. CART*+*L. chagasi*-ELISA	90.1 (81.0–96.0)	89.5 (75.2–97.0)	95.2 (91.4–99.0)	94.1 (86.0–98.4)
1. CART*+IFAT	92.5 (87.0–96.3)	71.0 (59.0–81.3)	94.0 (90.2–97.1)	86.0 (79.2–91.2)
2. CART*+IFAT	97.2 (92.2–100)	76.3 (60.0–89.0)	95.0 (89.0–100)	88.5 (79.2–95.0)
1. CART*+rK39-ELISA	98.0 (93.5–99.5)	88.4 (78.4–95.0)	97.2 (95.0–100)	94.2 (89.0–97.5)
2. CART*+rK39-ELISA	96.0 (88.1–99.1)	89.5 (75.2–97.1)	93.4 (87.0–100)	94.4 (86.4–98.5)
1. CART*+DAT	90.2 (84.0–95.0)	97.1 (90.0–100)	98.0 (96.1–99.5)	98.4 (94.2–100)
2. CART*+DAT	91.5 (82.5–97.0)	92.1 (79.0–98.3)	94.0 (87.3–100)	96.0 (88.0–99.1)
1. CART*+rK39 rapid test	94.0 (88.5–97.4)	98.5 (92.2–100)	99.0 (98.0–100)	99.2 (96.0–100)
2. CART*+rK39 rapid test	90.1 (81.0–96.0)	97.4 (86.2–100)	97.3 (95.0–100)	98.5 (92.0–100)

1 Test sample; 2 Validation sample.

*The CART model was composed by variables: Splenomegaly, leukopenia, cough, age and weight loss.

**Figure 2:**
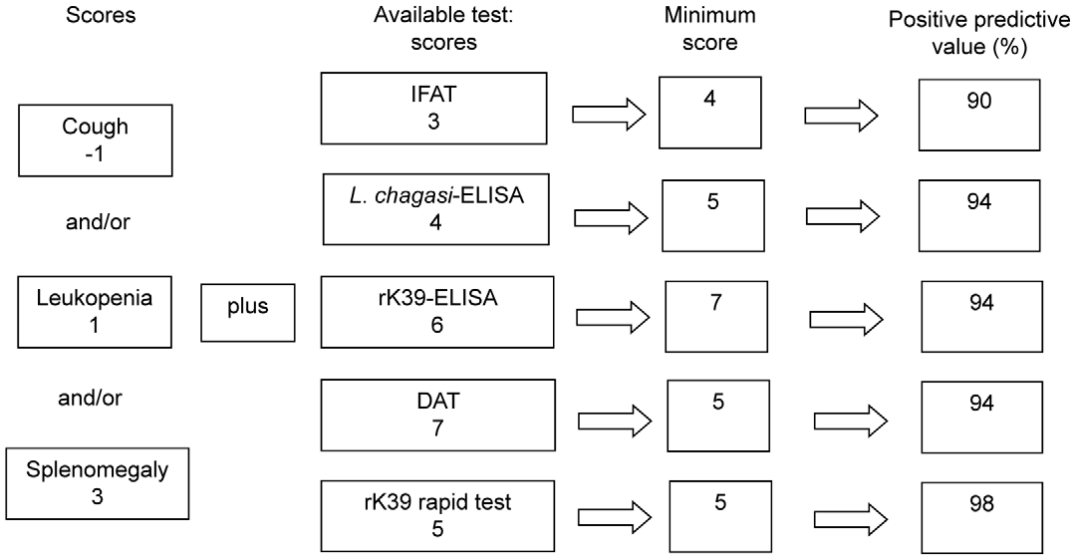
Example, based in models developed using logistic regression, on how a chart could be used.

Figure 2 presents one example, based in models developed using logistic regression, on how a chart could be used to help health professionals with the tests interpretation and the physicians with the clinical decision. In the validation sample, in the first model, composed only by clinical-laboratory variables, patients with score ≥3 (82/107–51%) showed a probability of having VL of 79% (data not shown). In the second model, when IFAT was added, patients with score ≥4 (78/107–73%) had a probability of VL of 90%. In the third model, which included clinical and laboratorial features and *L. chagasi* ELISA, patients with a score ≥5 (68/107–63%) had 94.1% probability of VL. In the model combining clinical-laboratory variables plus rK39 ELISA, patients with score ≥7 (72/107–67%) presented 94.4% VL probability. In the fifth model, adding DAT to clinical and laboratorial findings, patients with score ≥5 (68/107–63%) showed also a 94% probability of a VL diagnostic. Still, in the sixth model (clinical-laboratory plus rK39 rapid test) patients with score ≥5 (65/107–61%) showed the higher VL probability (PPV 98.5%).

## Discussion

VL is a serious disease, with repeatedly recognition of the lack of sufficient means for its elimination. Rapid diagnostic and adequate treatment of cases would certainly help to reduce morbidity and mortality and it may contribute also to decrease transmission where anthroponotic VL transmission occurs. Clinical diagnosis of VL is inaccurate because it's clinical presentation shares common features to several other diseases and can vary in different endemic areas. In the present study, splenomegaly, leukopenia and cough were the clinical-laboratory variables that remained in the predictive model using logistic regression; and splenomegaly, leukopenia, cough, age and weight loss were the clinical-laboratory variables that remained in the predictive model using CART for VL diagnosis.

Splenomegaly is a classic sign of VL that with the advance of disease can cause abdominal distension and pain. In the study by Tanoli et al. (2005) [14] in Pakistan, 95% of patients had splenomegaly and in the study by Daher et al. (2008) [15] and Rocha et al. (2011) [16] in Brazil, 96% and 94% of the patients, respectively, showed this signal, as well. Leukopenia and weigh loss are reported frequently in clinical studies involving patients with VL. In the study by Dursun et al. (2009) [17] in Turkey, 74% of the patients had leukopenia, and in the study by Queiroz et al. (2004) [18] and Daher et al. (2008) [15] in Brazil, 85% of the patients showed leukopenia and 95% showed weigh loss, respectively. Several authors have reported that the VL is predominant in children early in life and is associated with high morbidity and high number of deaths [17]–[18]. Other manifestations can be seen less consistent with the LV, such as cough and diarrhea [16], [18]. In this study cough was a sign negatively correlated to LV. Therefore, VL should be suspected in endemic areas when patients present enlarged spleen, leukopenia and weigh loss, especially in children early in life.

Laboratory diagnosis of VL is, still now, complex. The sensitivity of parasitological tests is suboptimal, ranging from 53–86% for bone marrow up to 93–99% for spleen aspirates [1]. Diagnostic research in VL has been damaged by the lack of a perfect gold standard. An alternative to the classical validation approach using parasitological diagnostic methods as the gold standard is the latent class analysis (LCA). LCA is based on the concept that the observed results of different imperfect tests for the same disease are influenced by a latent common variable, the true disease status, which cannot be directly measured [19]–[21]. Several studies used LCA methodology for the evaluation of diagnostic tests for VL, such as Boelaert et al. (1999, 2004 and 2008) [22]–[24], Horst et al. (2009) [25] and Menten et al. (2008) [26].

Less invasive methods are being evaluated for VL diagnosis. IFAT, ELISA, and a polymerase chain reaction are examples of these efforts. Unfortunately, all of these tests require laboratory infrastructure and specialized professionals. More recently, alternatives to the methods mentioned above, such as DAT and rapid test have become available. DAT and rK39 show high sensitivity, specificity, rapid results and are easy to use [5]–[7]. In the multicenter study performed in Brazil, which served as the basis for the development of the predictive models presented, the IFAT showed sensitivity of 88% and specificity of 81%, the *L. chagasi* ELISA showed sensitivity of 92% and specificity of 77%, the rK39-ELISA showed sensitivity of 97% and specificity of 84% [9], the rapid test IT-LEISH® showed sensitivity of 93% and specificity of 97% and the DAT showed sensitivity of 90% and a specificity of 96% [7].

In the present study, it was possible to generate predictive models for VL with good general predictive performance. It was observed that the generated models showed better performance compared to the model based only on clinical-laboratory variables, reinforcing the importance of diagnostic tests in patients' management. From the standpoint of performance and practicality, the sixth model, composed of clinical-laboratory variables and the rK39 rapid test, developed using both logistic regression and CART, may represent the best suitability for use in peripheral services and referral centers, since the rapid test is easy to perform and to interpret, with result available within 20 minutes. Other models, such as the second, composed of clinical-laboratory variables and the IFAT could be useful in services that have this technique already implemented.

Clinical prediction models have been developed to help physicians improve the assessment of an individual's risk of a disease or to predict an outcome, for a great number of diseases, such as tuberculosis and pneumonia. It is the first time that this type of predictive model is developed for human VL and it represents an innovative approach in disease diagnosis. It was out of the scope of this study to evaluate the interference of epidemics or the seasonality of the disease and the possible use of other models as the early warning systems (EWS) based on environmental variables that have been developed to predict the occurrence of epidemics of cutaneous leishmaniasis and could be also applied to VL [27].

The use of a control group (non-VL patients) with a variety of diseases that can mimic VL and representative of the population that seeks references centers for VL in Brazil is one of the strong features of this study, providing a realistic scenario for the use of the predictive models generated. However, there are also some methodological limitations in our study that should be considered before deciding to apply the results of models in clinical practice. First, although our modeling strategy used geographically different samples for deriving and validating the models, one need to be cautious about the possibility that the patients enrolled in our study may not be representative of populations from other settings. Second, the patients were already identified at admission to be at risk for VL, in this sense our models were developed for a population attending to referral centers and might not be useful in different circumstances. Third, the small size of the validation sample, as compared to sample derivation contributed to the relatively low precision of sensitivity, specificity, PPV and the area under the receiver operator characteristic estimates in the validated models. Fourth, the use of leukopenia as a predictor might impair the use of such models in many endemic areas where a complete blood count is difficult to be performed. Unfortunately, a model without leucopenia did not performed well in our sample. Therefore, the development of simpler models with good predictive performance in settings where blood counts are not readily available is a challenge that should be explored in other studies.

The scoring system derived from logistic regression and the classification scheme based on CART models are simple and based on the clinical-laboratory findings that are easily available in most clinical settings. The model composed of clinical-laboratory variables and the rK39 rapid test developed using both logistic regression and the model CART showed the best performance and it could be used in health services. This assessment tool could support a physician's decision but should not preclude his assistance.
